# Altered intestinal barrier contributes to cognitive impairment in old mice with constipation after sevoflurane anesthesia

**DOI:** 10.3389/fnut.2023.1117028

**Published:** 2023-09-12

**Authors:** Tianyun Zhao, Junming Lu, Jingwen Qin, Yanxin Chen, Ziwen Shi, Wei Wei, Peng Xiong, Daqing Ma, Xingrong Song

**Affiliations:** ^1^The First Affiliated Hospital of Jinan University, Guangzhou, China; ^2^Department of Anesthesiology, Guangzhou Women and Children’s Medical Center, Guangzhou Medical University, Guangzhou, China; ^3^Department of Anesthesiology, The Second Affiliated Hospital of Guangzhou University of Chinese Medicine, Guangzhou, China; ^4^Department of Public Health and Preventive Medicine, School of Medicine, Jinan University, Guangzhou, China; ^5^Division of Anaesthetics, Pain Medicine & Intensive Care, Department of Surgery & Cancer, Faculty of Medicine, Imperial College London, Chelsea and Westminster Hospital, London, United Kingdom; ^6^National Clinical Research Center for Child Health, Zhejiang, China; ^7^Guangdong-Hong Kong-Macau Institute of CNS Regeneration, Ministry of Education CNS Regeneration Collaborative Joint Laboratory, Jinan University, Guangzhou, China

**Keywords:** perioperative neurocognitive dysfunction, gastrointestinal dysfunction, slow transit constipation, gut permeability, microglia

## Abstract

**Background:**

Elderly patients have a high risk of developing postoperative cognitive dysfunction (POCD). Gastrointestinal disorders, such as constipation, in the elderly population may be involved in the pathogenesis of neurological disorders by promoting inflammatory responses due to a ‘leaky gut’. General anesthetic sevoflurane may impair gastrointestinal function in elderly patients to trigger neurological complications following surgery. Therefore, we hypothesized that elderly individuals with gastrointestinal dysfunction may be more vulnerable to sevoflurane and consequently develop POCD.

**Methods:**

Aged mice were randomly divided into four groups: control (CTRL), CTRL+sevoflurane (Sev), slow transit constipation (STC), and STC + Sev. Mice in the STC and STC + Sev groups were intra-gastrically administrated loperamide (3 mg/kg, twice a day for 7 days) to induce a slow transit constipation (STC) model determined with fecal water content and the time of first white fecal pellet, whereas mice in the other groups received the similar volume of saline. One week later, mice in the CTRL+Sev group and STC + Sev group received 2% sevoflurane for 2 h. The gut permeability evaluated with 4-kDa fluorescein isothiocyanate (FITC)-dextran, serum cytokines, microglia density, TLR4/NF-κB signaling expression, and POCD-like behavioral changes were determined accordingly.

**Results:**

The loperamide-induced STC mice had decreased fecal water content and prolonged time of first white fecal pellet. Sevoflurane exposure caused significantly increased gut permeability and serum cytokines, as well as the activation of microglia and the TLR4/NF-κB signaling pathway in the prefrontal cortex of the aged STC mice. Sevoflurane also caused cognitive impairment and emotional phenotype abnormality in aged STC mice.

**Conclusion:**

Aged STC mice were more vulnerable to sevoflurane anesthesia and consequently developed POCD-like behavioral changes. Our data suggest that gastrointestinal disorders including constipation may contribute to the development of POCD.

## Background

Due to the aging society, more elderly patients are undergoing anesthesia and surgery. Postoperative neurological complications including acute postoperative delirium (POD) and postoperative cognitive dysfunction (POCD) are common following surgery (1). All these complications cause longer hospitalization, higher economic and social burdens, and lead to more deaths. Furthermore, patients with POCD may have a high risk of exacerbation towards the development of Alzheimer’s disease (2). Therefore, understanding the aetiologic mechanism of POCD is urgently needed for developing preventive and treatment strategies. Recently, growing evidence demonstrated that gastrointestinal dysfunction including constipation by the promotion of inflammatory responses due to a ‘leaky gut’ and microbial translocation may be involved in the development of autoimmune diseases, neurodegenerative diseases (such as Alzheimer’s disease and Parkinson’s disease) and neuropsychiatric disorders (such as depression and autism spectrum disorders) (3–7). The prevalence of constipation in the general population is approximately 20%, increasing with age, and more frequent in hospital inpatients, and even up to 60–80% in residents in long-term care facilities (8–10). Constipation, one of the most common gastrointestinal disorders, is defined by symptoms including excessive straining, a sense of incomplete evacuation, failed or lengthy attempts to defecate, abdominal bloating, and hard consistency of stools. Constipation is classified into two types: primary and secondary. Primary constipation can be further classified into functional defecation disorder, slow-transit constipation, and constipation-predominant irritable bowel syndrome, which is due to inadequate propulsive forces in the colon (11). Anesthesia and surgery may have further detrimental effects on patients with constipation who may have gut barrier permeability changes and may exaggerate inflammatory responses during the perioperative period. However, this warrants further study.

The clinical symptoms of POCD include disorientation, impaired memory, and abstract thinking disorder, accompanied by cooperation and impaired social activities. Although the underlying mechanisms of POCD remain largely unknown, systemic inflammation and subsequent neuroinflammation have been well documented to be one of the mechanisms that are responsible for the development of such neurological complications following surgery (12). General anesthetics especially a commonly used inhalational anesthetic, sevoflurane, may also be a contributor to the development of POCD by promoting microglial activation and neuroinflammation (13). However, most studies have concentrated on the hippocampus critically involved in memory and cognition, and very few studies have investigated the prefrontal cortex (PFC), which is responsible for social, cognitive, and emotional fucntions that are commonly disrupted in neuropsychiatric diseases (14). It is well known that gut barrier integrity is crucial for preventing the translocation of hostile antigens, environmental toxins, and pollutants into interepithelial and subepithelial tissue and blood (6, 7). In addition, the gastrointestinal key functions, including epithelial permeability integrity, are governed by autonomic innervation and the intrinsic enteric nervous system (15, 16). Therefore, it is proposed that general anesthetics such as sevoflurane may act on nervous systems and in turn affect intestinal permeability, thus causing systemic inflammation and leading to cognitive dysfunction. We, therefore, hypothesized that, except for inflammatory responses induced by surgical trauma, gastrointestinal dysfunction conditions, such as constipation, which was reported to have an increased intestinal permeability and cause systemic immune response (17), may also contribute to systemic inflammatory response and postoperative neurological complications. The present study was, therefore, designed to investigate in a constipation model in mice whether gastrointestinal disorders, such as constipation, are more vulnerable to sevoflurane exposure, which subsequently increases gastrointestinal permeability in triggering systemic inflammation and, finally, impairing brain functions.

## Methods and materials

### Ethics statement and experimental animals

All experimental protocols were carried out in accordance with Institutional guidelines (Animal Care and Use Committee of Jinan University, Guangzhou, China). Efforts were made to minimize the overall number of sacrificed animals and their suffering from experiments. Male C57BL/6 mice that were 16 months old were purchased at the Guangdong Medical Laboratory Animal Center. They were raised in standard cages under light- and temperature-controlled conditions (22 ± 2°C, 12-h light/dark cycle) and received food and water *ad libitum*.

### Animal study

#### Constipation model and sevoflurane exposure procedure

After 1 week of acclimation to the laboratory conditions, the mice were randomly divided into four groups (*n* = 47) as follows: control group (CTRL), loperamide-induced slow transit constipation (STC)-model group (STC), CTRL+sevoflurane exposure group (CTRL+Sev), and STC + sevoflurane exposure group (STC + Sev). The constipation model was established by intragastric administration of loperamide (3 mg/kg body weight, twice a day) for 7 days as previously reported, with minor modifications (18). The mice in the CTRL group were given the same volume of normal saline by intragastric administration. One week after the constipation model was established, mice of the CTRL+Sev group and STC + Sev group were exposed to 2% sevoflurane (Hengrui Medicine Co. Ltd., Jiangsu, China), which is the clinically relevant concentration of sevoflurane (the minimum alveolar concentration of sevoflurane in aged mice is 1.52%) (19). Sevoflurane was delivered with 30% oxygen balanced with nitrogen at a flow of 1 L/min for 2 h in a 16 × 23 × 12 cm plexiglas chamber. The concentration of gas was continuously monitored (BeneView T8, Mindray Bio-Medical Electronics Co. Ltd., Shenzhen, China) in the outlet of the chamber and sevoflurane was started with 4.0% for the first 15 min to allow equilibration and then was gradually decreased to 2.0% for maintenance. Mice of the CTRL group and STC group were supplied with identical gases without sevoflurane. Mice had been fasted for 4 h before gas exposure and returned to their cages when they fully woke up and moved freely.

#### Measurements

**Figure 1:**
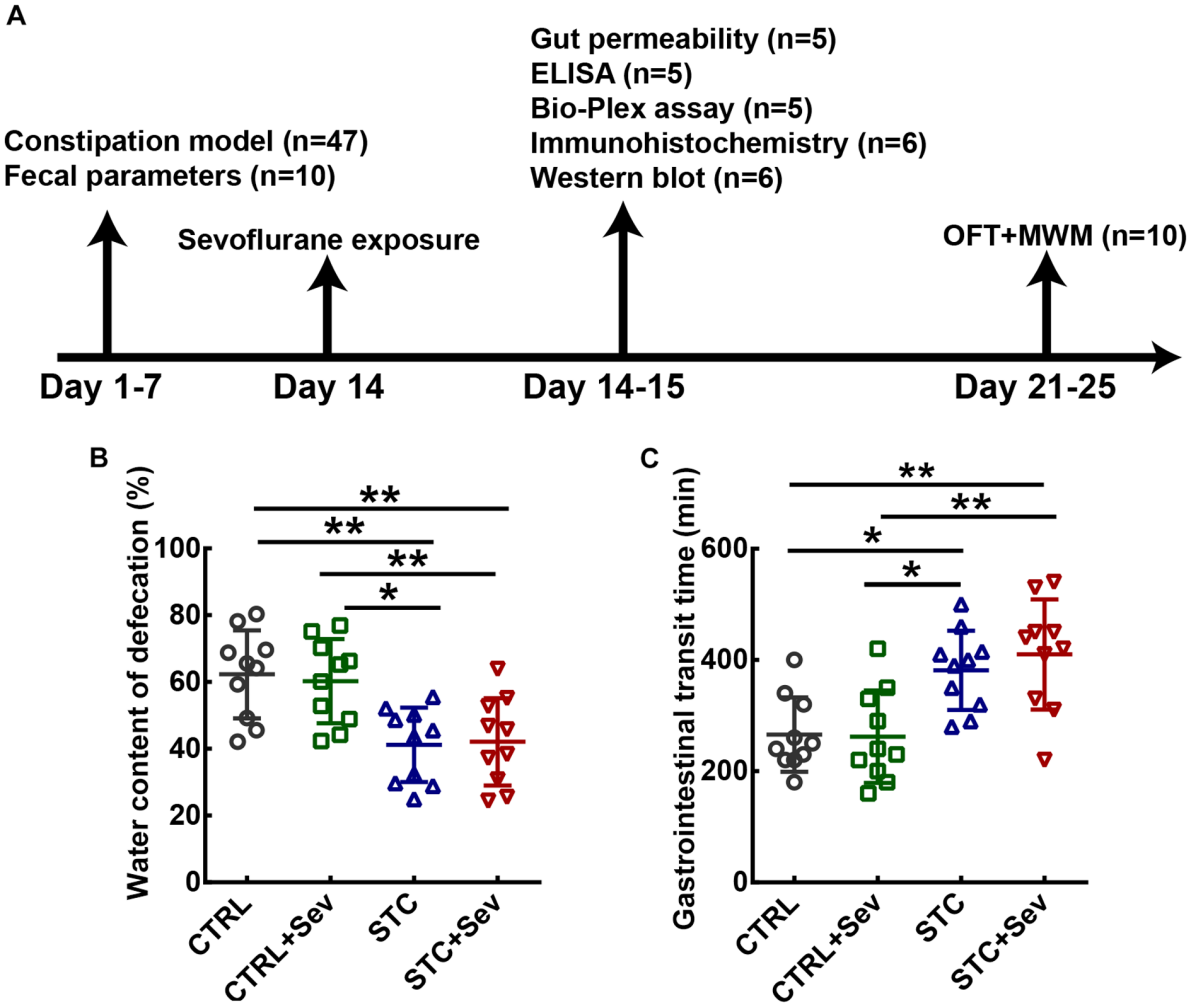
The flow chart of the experimental protocols and establishment of loperamide-induced STC demonstrated by fecal characteristics. **(A)** Schematic presentation of loperamide-induced STC mice model establishment and sevoflurane treatment. **(B)** Quantitative analysis of the fecal water content (*n* = 10 mice/group). **(C)** Quantitative analysis of the time for expulsion of the first white fecal pellet (*n* = 10 mice/group). Data were presented as mean ± SD. One-way analysis of variance (ANOVA) with Tukey’s *post hoc* comparison. ^*^p < 0.05; ^**^p < 0.01. CTRL, control; Sev, sevoflurane exposure; STC, slow transit constipation; ELISA, Enzyme-Linked Immunosorbent Assay; MWM, Morris water maze test; OFT, open field test.

The parameters used to determine the successful establishment of the STC model included the water content of feces (*n* = 10) performed on day 0 and day 8 and gastrointestinal transit (*n* = 10) performed on day 14 (Figure 1A). Gut permeability (*n* = 5) was assessed with intragastric administration of 4-kDa FITC dextran after sevoflurane exposure (on day 14) at different time points (2, 6, 12, and 24 h). Blood samples (*n* = 5) were collected from the portal vein 4 h after sevoflurane anesthesia to determine the level of plasm inflammatory cytokines using Bio-Plex assay, and the concentrations of plasm endotoxin (ET), known as lipopolysaccharide (LPS), intestinal fatty acid-binding protein (I-FABP), and diamine oxidase (DAO) using enzyme-linked immunosorbent assay (ELISA) kits. After 24 h of sevoflurane exposure, mice were randomly selected to harvest brain tissue under terminal anesthesia for Western blotting (n = 6) and histological assessments (*n* = 6). The other cohort mice (*n* = 10) were used for behavioral assessments (*n* = 10) conducted 2 weeks after sevoflurane exposure.

#### Fecal parameters and gastrointestinal transit

The feces samples were collected before (day 0) and 8 days (day 8) after intragastric administration of loperamide. The wet weight of the same number of fecal particles from each mouse was measured and then dried in the oven at 60°C for 3 h to obtain the dry weight. The water content of feces (%) was calculated with the following formula: Water content (%) = (Wet weight -Dry weight)/Wet weight × 100%. One week after the end of the loperamide administration, a test for gastrointestinal transit was performed as previously described with minor modification (20). After fasting 12 h with free access to water, 150% (w/v) Barium sulfate (BaSO4) was administered by gavage. Fecal pellets were monitored to obtain the first white pellet out time.

#### Gut permeability

Gut permeability was assessed with intragastric administration of 4-kDa FITC dextran (500 mg/kg, 100 mg/mL) after sevoflurane exposure at different time points (2, 6, 12, and 24 h). To avoid the interferences of intestinal contents, mice were fasted for 5 h prior to the measurements. Blood was collected from the portal vein 1 h after intragastric administration of FITC dextran and centrifugated at 3000xg for 5 min to prepare plasm for measuring FITC-dextran concentration with a fluorescence spectrophotometer (Perkin Elmer Singapore Pte. Ltd., Singapore) at the excitation wavelength of 485 nm and the emission wavelength of 520 nm. Standard curves for calculating the FITC-dextran concentration in the samples were prepared by diluting FITC-dextran in plasm.

#### Enzyme-linked immunosorbent assay (ELISA)

The concentrations of LPS, intestinal fatty acid-binding protein (I-FABP), and diamine oxidase (DAO) were determined using enzyme-linked immunosorbent assay (ELISA) kits according to the manufacturer’s instructions (LPS: JYM0588Mo; I-FABP: JYM0809Mo; DAO: JYM0138Mo; Genomei Biotechnology Co., LTD, Huhan, China). Blood samples were collected from the portal vein 4 h after sevoflurane anesthesia and plasm samples were prepared to measure LPS, I-FABP, and DAO accordingly with each respective kit through fluorescence spectrophotometer (Perkin Elmer Singapore Pte. Ltd., Singapore). Standard curves were plotted, and then the concentration in each sample was calculated.

#### Bio-Plex assay

Blood samples were collected from the portal vein 4 h after sevoflurane anesthesia and inflammatory cytokines in plasm were determined using Bio-Plex Pro Mouse Cytokine 8-plex Assay (M60000007A, Bio-Rad, Hercules, CA, United States) according to manufacturer’s instructions.

#### Western blot

Tissues from the prefrontal cortex (PFC) (*n* = 6/group) were dissociated in lysis buffer containing protease inhibitors on ice for 30 min and homogenized via ultrasonication (Ningbo scientz biotechnology CO. LTD, Ningbo, China). After centrifugation at 12,000 g for 10 min at 4°C, supernatants were obtained and protein concentrations were measured with a BCA assay kit (Beyotime Institute of Biotechnology, China). A total of 30 μg protein samples were loaded in SDS-PAGE gels and run at room temperature, followed by transfer to polyvinylidene fluoride membranes. After blocking for 1 h in 5% milk in PBS or tris-buffered saline and 5% bovine serum albumin, membranes were incubated with mouse anti-Toll-like receptor 4 (TLR4) (1:250, Cat.No.sc-293,072, Santa Cruz Biotechnology, Santa-Cruz, United States), rabbit anti-nuclear factor-κB (NF-κB) (1:1000, Cat.No.06–418, Millipore, United States), rabbit anti-active-NF-κB (1:1000, Cat.No.MAB3026, Millipore, United States), rabbit anti-GAPDH (1:1000, Cat.No.5174, Cell Signaling Technology, United States) overnight at 4°C. Signal was detected using HRP-conjugated rabbit or mouse secondary antibodies followed by chemiluminescence using a Super Signal West Pico kit (Pierce) and Hyper film ECL (Amersham Biosciences). All experiments were carried out at least in triplicate. The expressions of TLR4 and active-NF-κB/NF-κB were determined by calculating their density ratio to the GAPDH band and then normalized to the control group.

#### Histology and immunohistochemistry

After 24 h of sevoflurane exposure, the brains of the mice were harvested under terminated anesthesia for paraffin sections. After dewaxing and antigen recovery, coronal paraffin sections (5 μm in thickness) of the prefrontal cortex (+ 2.96 mm from bregma and + 2.46 mm from interaural landmark) and dorsal hippocampus (−1.70 mm from bregma and − 2.18 mm from interaural landmark) were incubated overnight with primary mouse anti-ionized calcium-binding adaptor molecule 1 (Iba-1) (1:500, abcam, Cat.No.MAB1572) at 4°C. Iba-1 signal was detected with a mouse-rabbit ABC kit (PK-6200, Universal, Vector). Images were captured using a camera system (SP8 and DMi8 DFC7000J, Leica, Germany). Iba-1 positive cells were counted using Image J (NIH, United States) and expressed as cell numbers/mm^**2**^.

### Behavioral tests

#### Open field test (OFT)

The open field test was used to assess the spontaneous locomotor activity and emotional status of rodents (21). Mice were placed gently in the center of a dimly lit open-field apparatus (50 cm × 50 cm × 40 cm) constructed with Plexiglas walls and a white floor and were allowed to move freely for 15 min 1 day after sevoflurane exposure. The arena was cleaned with 70% ethanol after each trial. Walking traces were recorded with a video camera and analyzed using the Noldus EthoVision (XT 7.1, Wageningen, Netherlands). The traveling distance, speed, and frequency of the center zone visits were calculated.

#### Morris water maze (MWM)

An iron circular tank of 120 cm in diameter and 80 cm in height was filled with water to a depth of 35 cm at 23 ± 1°C and divided into four equal quadrants with high-contrast, external cues mounted on the surrounding walls (geometrical patterns of star, square, triangle, and roundness). An escape platform (5× 5 cm^**2**^) was submerged 0.8 cm below the water surface in the center of one quadrant. Swimming trajectories were recorded using a video camera by a computerized system (Noldus EthoVision XT 7.1). Procedures included adaptive training on the first day, subsequently placed trials for continuous 3 days, and then the probe trials on the fifth day.

Parameters including mean velocity, mean escape latency in the placed trials, and the frequency of crossing the platform in the probe trials were recorded and analyzed.

### Statistical analysis

Data were presented as dot plots and mean ± standard deviation (SD) and analyzed with GraphPad Prism 6.0 (GraphPad Software, Inc., La Jolla, CA, United States). Body weight and plasm 4-kDa FITC dextran concentration were analyzed using a two-way ANOVA with repeated measures. Followed by Bonferroni’s *post hoc* tests for comparison. Other treatment effects were analyzed by one-way ANOVA followed by Bonferroni’s *post hoc* tests for comparison when normality (and homogeneity of variance) assumptions were satisfied, otherwise, the Kruskal-Wallis test was applied to analyze the differences between groups followed by Dunn’s multiple comparison test. A *p value* < 0.05 was considered to be of statistical significance.

## Results

### Establishment of loperamide-induced STC model in aged mice

To investigate the influence of sevoflurane on the constipation mouse model, we established a slow transit constipation (STC) model *via* oral gavage administration of loperamide, and sevoflurane was administrated 1 week after the STC model was established (see Figure 1A). The fecal water content was used to evaluate defecation status. One-way ANOVA indicated that the water content of fecal pellet in the STC and STC + Sev groups was significantly lower than that of the CTRL and CTRL+Sev groups (Figure 1B, *n*= 10, *p* < 0.01) on the 8^th^ day of loperamide administration. In addition, the gastrointestinal transit, determined by measuring the time for expulsion of the first white fecal pellet, was significantly longer in STC (382 ± 71 min) and STC + Sev (410 ± 99 min) mice compared to CTRL (266 ± 67 min) and CTRL+Sev (262 ± 83 min) mice (Figure 1C, *n*= 10, *p* < 0.01). These data suggested the successful establishment of the constipation mice model. The body weights in the STC and STC + Sev groups were less than those of the CTRL and CTRL+Sev groups but did not reach statistical significance (data not shown).

### Aged STC mice after sevoflurane exposure experienced greater detrimental effects on gut permeability and intestinal injury

**Figure 2:**
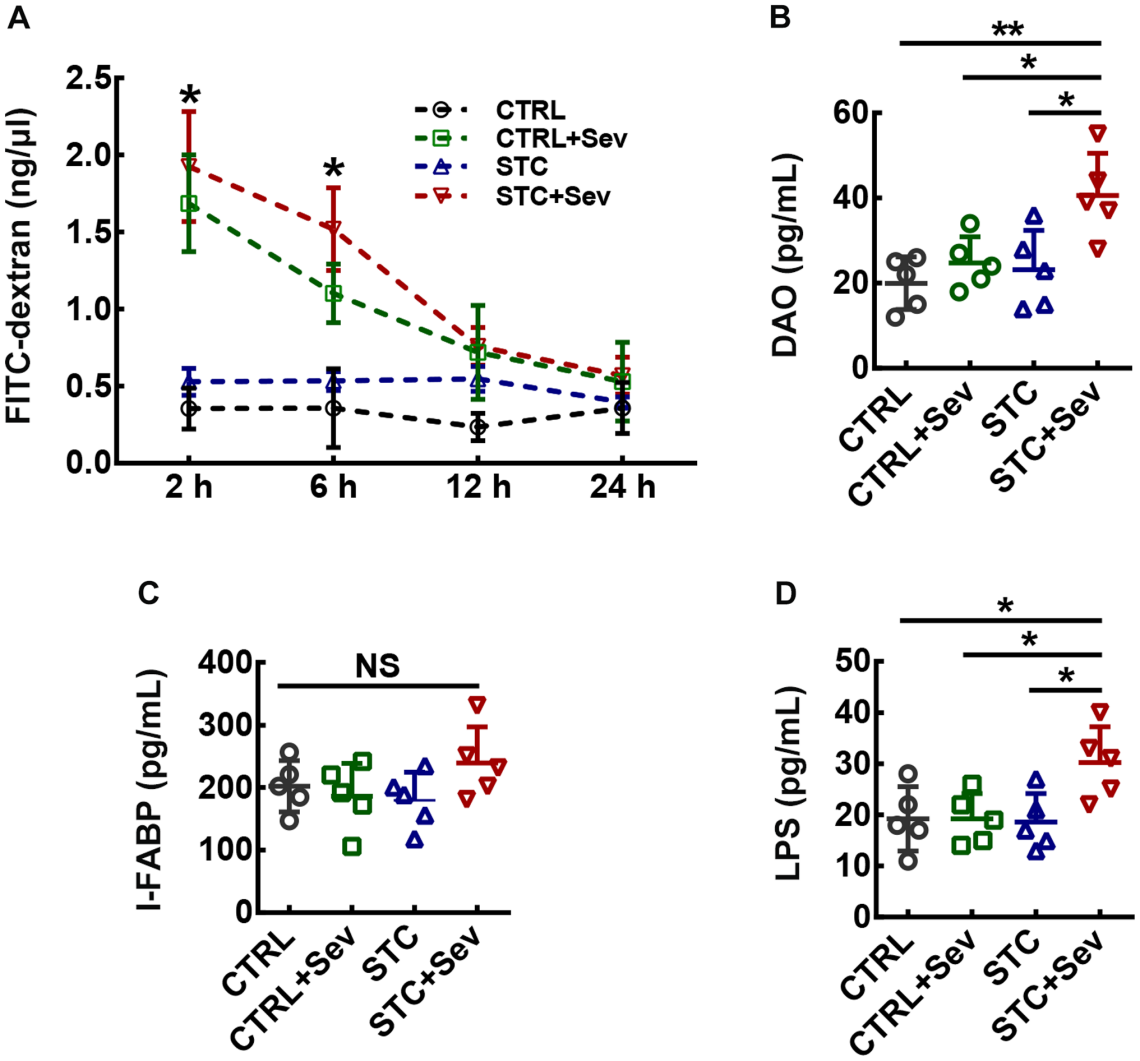
Aged STC mice after sevoflurane exposure experienced greater detrimental effects on gut permeability and intestinal injury. **(A)** Statistic analysis of the plasm 4-kDa FITC dextran concentration at 2, 6, 12, and 24 h after intragastric administration (two-way ANOVA with repeated measures, F_interaction(9, 64)_ = 11.38, *p* < 0.01; F_time(3, 64)_ = 39.55, *p* < 0.01; F_treatment(3, 64)_ = 73.23, *p* < 0.01). **(B–D)** Statistical analysis of the plasm concentration of DAO (B, *n* = 5, One-way ANOVA, F_3,16_ = 6.51, *p* < 0.01), I-FABP (C, *n* = 5, one-way ANOVA, F_3,16_ = 1.46, *p* = 0.26) and LPS (D, *n* = 5, One-way ANOVA, F_3,16_ = 4.34, *p* = 0.02) at 4 h after sevoflurane anesthesia. Data were presented as mean ± SD. ^*^p < 0.05; ^**^p < 0.01. CTRL: control; Sev: sevoflurane exposure; STC: slow transit constipation.

To examine whether the intestinal barrier integrity would be more vulnerable to sevoflurane exposure damage in the aged STC mice, the gut permeability was assessed by permeability to a 4 kDa FITC-dextran. Plasm 4-kDa FITC dextran concentration in four groups was gradually decreased from 2 h after intragastric administration of 4 kDa FITC-dextran. A two-way ANOVA with repeated measures revealed that plasm 4-kDa FITC dextran concentration in the STC + Sev and CTRL+Sev groups were significantly higher compared to that of the CTRL and STC groups at 2 h and 6 h after intragastric administration, and the concentration in the STC + Sev group was significantly higher than that of the CTRL+Sev mice (Figure 2A, F_interaction(9, 64)_ = 11.38, *p* < 0.01; F_time(3, 64)_ = 39.55, *p* < 0.01; F_treatment(3, 64)_ = 73.23, *p* < 0.01). The intestinal barrier includes surface mucus, epithelial layer, and immune defenses, and intestinal injury, such as direct epithelial damage, disrupts intestinal barrier function, and consequently causes gut permeability and increased level of plasm endotoxin (22). Therefore, typical epithelial injury markers of I-FABP, DAO, and plasm LPS were chosen to evaluate the intestinal barrier function 4 h after the sevoflurane exposure. One-way ANOVA analysis revealed the concentration of DAO was significantly higher in STC + Sev mice compared to the mice of the other three groups, but there were no significant differences between these three groups mice (Figure 2B, F_3,16_ = 6.51, *p* < 0.01). In addition, there was no significant difference in the plasm concentration of I-FABP between the four groups (Figure 2C, F_3,16_ = 1.46, *p* = 0.26). Furthermore, the concentration of LPS was significantly higher in STC + Sev mice compared to mice in the other three groups while there were no significant differences between these three groups mice (Figure 2D, F_3,16_ = 4.34, *p* = 0.02).

### Sevoflurane exposure increased serum concentration of inflammatory cytokines in aged STC mice

**Figure 3:**
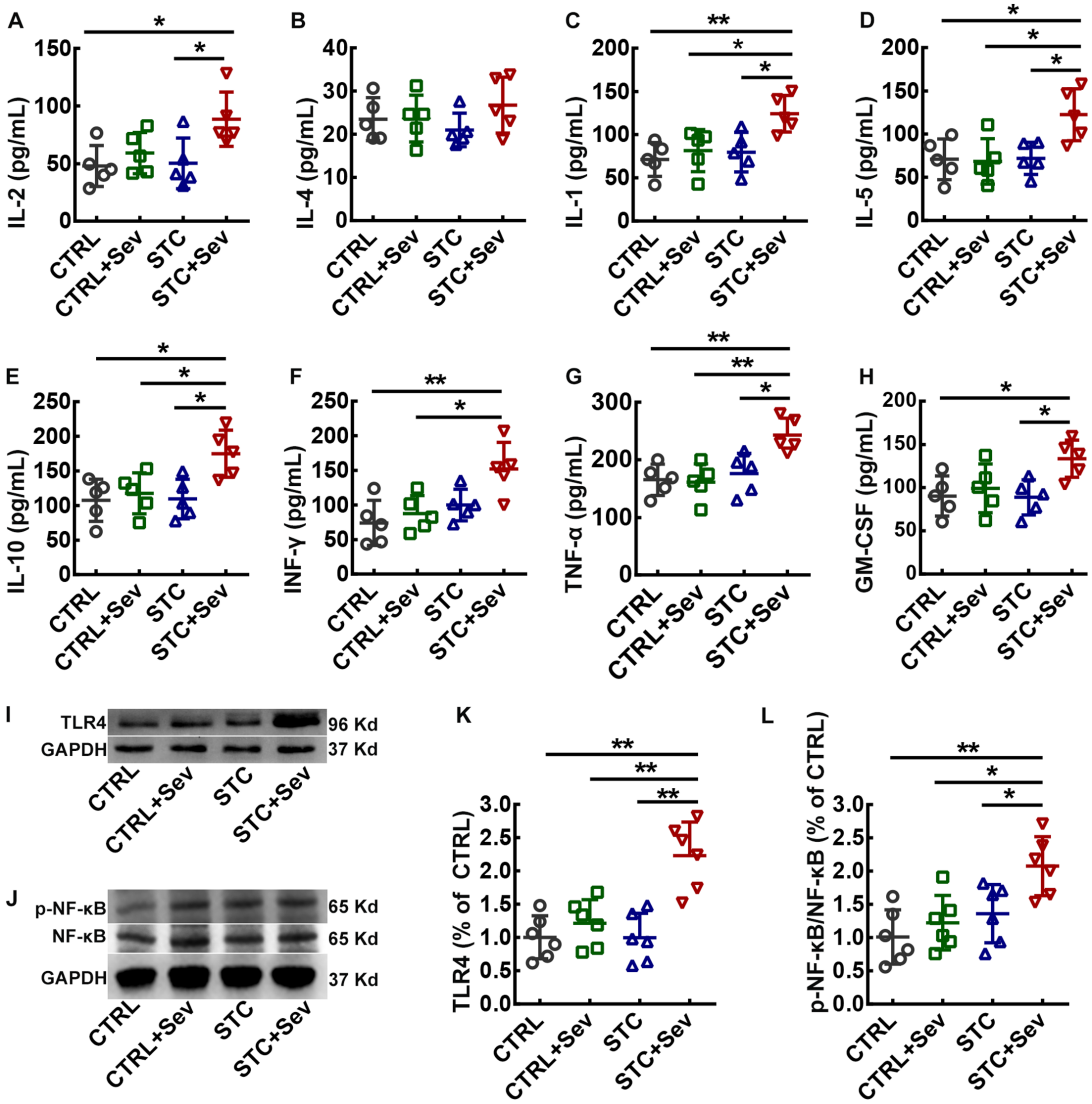
Sevoflurane exposure increased the serum concentration of inflammatory cytokines and activated the TLR4/NF-κB signaling pathway of the PFC in aged STC mice. **(A–H)** Statistical analysis of the concentrations of IL-2 (A, F_3,16_ = 4.16, *p* = 0.02), IL-4 (B, F_3,16_ = 1.00, *p* = 0.42), IL-1 (C, F_3,16_ = 5.77, *p* < 0.01), IL-5 (D, F_3,16_ = 5.48, *p* < 0.01), IL-10 (E, F_3,16_ = 5.42, *p* = 0.01), INF-γ (F, F_3,16_ = 6.28, *p* < 0.01), TNF-α (G, F_3,16_ = 7.58, *p* < 0.01), and GM-CSF (H, F_3,16_ = 3.96, *p* = 0.03) at 4 h after sevoflurane anesthesia. **(I)** Representative western blot bands for TLR4 and GAPDH proteins. **(J)** Representative western blot bands for active-NF-κB, NF-κB, and GAPDH proteins. **(K)** Quantitative analysis of the expression of TLR4 at 24 h after sevoflurane anesthesia (*n* = 6, F_3,16_ = 13.37, *p* < 0.01). **(L)** Quantitative analysis of the relative expression of active-NF-κB/NF-κB at 24 h after sevoflurane anesthesia (*n* = 6, F_3,16_ = 7.04, *p* < 0.01). Data were presented as mean ± SD. One-way analysis of variance (ANOVA) with Tukey’s *post hoc* comparison. ^*^ *p* < 0.05; ^**^p < 0.01. CTRL, control; Sev, sevoflurane exposure; STC, slow transit constipation.

Endotoxin leaking from the injured intestinal barrier might lead to gut-derived endotoxin translocation in circulating and activate systematic inflammatory responses, resulting in increased cytokines in blood circulation. Therefore, we measured inflammatory cytokines in the serum collected from inferior ven cv. to evaluate the inflammatory cytokines in blood circulation. As shown in Figure 3, there were no significant differences between IL-2 (Figure 3A, F_3,16_ = 4.16, *p* = 0.02) and IL-4 (Figure 3B, F_3,16_ = 1.00, *p* = 0.42) between the CTRL+Sev and the STC + Sev groups, although the concentrations of IL-2 in the STC + Sev group was significantly higher than that of the CTRL group. Besides, the concentrations of cytokines including IL-1 (Figure 3C, F_3,16_ = 5.77, *p* < 0.01), IL-5 (Figure 3D, F_3,16_ = 5.48, *p* < 0.01), IL-10 (Figure 3E, F_3,16_ = 5.42, *p* = 0.01), INF-γ (Figure 3F, F_3,16_ = 6.28, *p* < 0.01), TNF-α (Figure 3G, F_3,16_ = 7.58, *p* < 0.01), and GM-CSF (Figure 3H, F_3,16_ = 3.96, *p* = 0.03) were significantly increased in the STC + Sev group mice comparing the rest of the three groups, whereas no significant differences were found among the CTRL, CTRL+Sev, and STC groups.

### Sevoflurane exposure activated the TLR4/NF-κB signaling pathway and upregulated expression of microglia in the prefrontal cortex and hippocampus of aged STC mice

**Figure 4:**
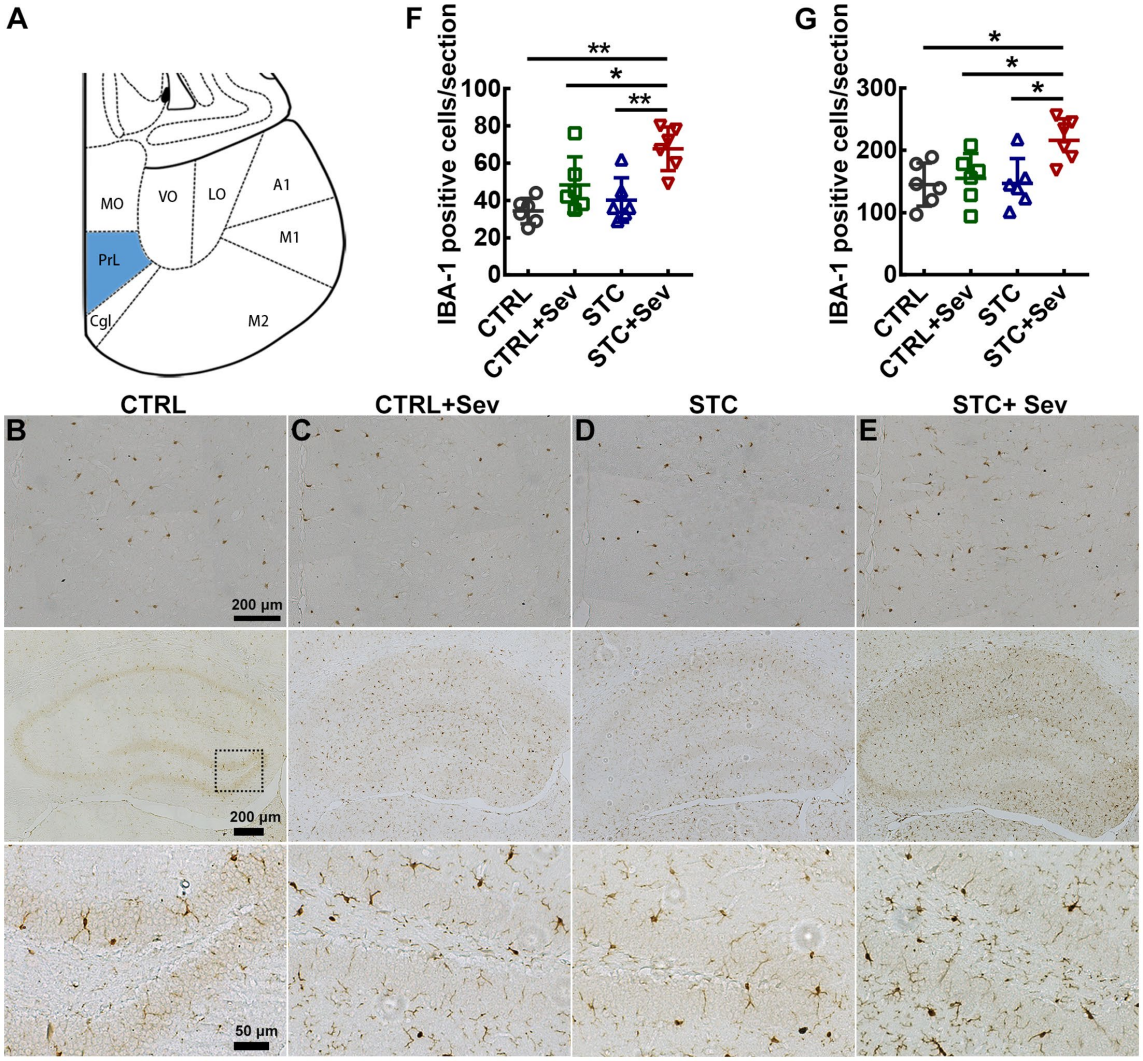
Sevoflurane exposure upregulated the expression of microglia in the prefrontal cortex and hippocampus of aged STC mice. **(A)** The schema indicated the mPFC (blue area) for images taken and analysis. **(B–E)** IBA-1-positive cells in the mPFC (top panel) and hippocampus (middle and bottom panel) in the CTRL **(B)**, CTRL+Sev **(C)**, STC **(D)**, and STC + Sev **(E)** groups mice at 24 h after sevoflurane anesthesia. **(F)** The number of microglia marked by IAB-1 immunostaining in the mPFC (*n* = 6, F_3,16_ = 9.19, *p* < 0.01). **(G)** The number of microglia in the hippocampus (*n* = 6, F_3,16_ = 4.97, *p* = 0.01). Scale bars were 200 μm in the top and middle panel of **B–E**, and 50 μm in the bottom panel of **B–E**. Data were presented as mean ± SD. One-way analysis of variance (ANOVA) with Tukey’s *post hoc* comparison. ^*^p < 0.05; ^**^p < 0.01. CTRL: control; Sev: sevoflurane exposure; STC, slow transit constipation.

To further investigate whether sevoflurane exposure activated the TLR4/NF-κB signaling pathway in the critical brain region of the PFC, the expression of TLR4 and relative expression of active-NF-κB/NF-κB were measured using western blot. As depicted in Figure 3, the expression of TLR4 (Figure 3K, F_3,16_ = 13.37, *p* < 0.01) and relative expression of active-NF-κB/NF-κB (Figure 3L, F_3,16_ = 7.04, *p* < 0.01) were significantly upregulated in aged STC mice when comparing with the other three groups. As previously reported, both sevoflurane exposures resulted in compromised blood–brain barrier (BBB) integrity and increased permeability (23). In addition, inflammatory cytokines themselves can induce BBB disruption and enhance the BBB permeability, and then peripheral harmful molecules including inflammatory cytokines leak into the brain and activate microglia, resulting in neuroinflammation (24). We chose two critical regions related to cognition and emotion including the medial prefrontal cortex (mPFC) and hippocampus to investigate the microglia activation. The number of microglia marked by IBA-1 immunostaining selected from the mPFC (Figure 4A, blue area) was significantly increased in the STC + Sev group compared to the rest of the three groups (Figures 4B–F, F_3,16_ = 9.19, *p* < 0.01). Similarly, a significantly increased number of microglia was observed in the hippocampus in the STC + Sev mice (Figures 4B–E,G, F_3,16_ = 4.97, *p* = 0.01). In addition, microglia morphology evidenced by the changes of the soma and branches in the STC + Sev mice of the PFC and hippocampus showed larger soma and shorter branches, which indicated an “activated state” compared to the other groups.

### Sevoflurane exposure caused cognitive and affective disorders in aged STC mice

**Figure 5:**
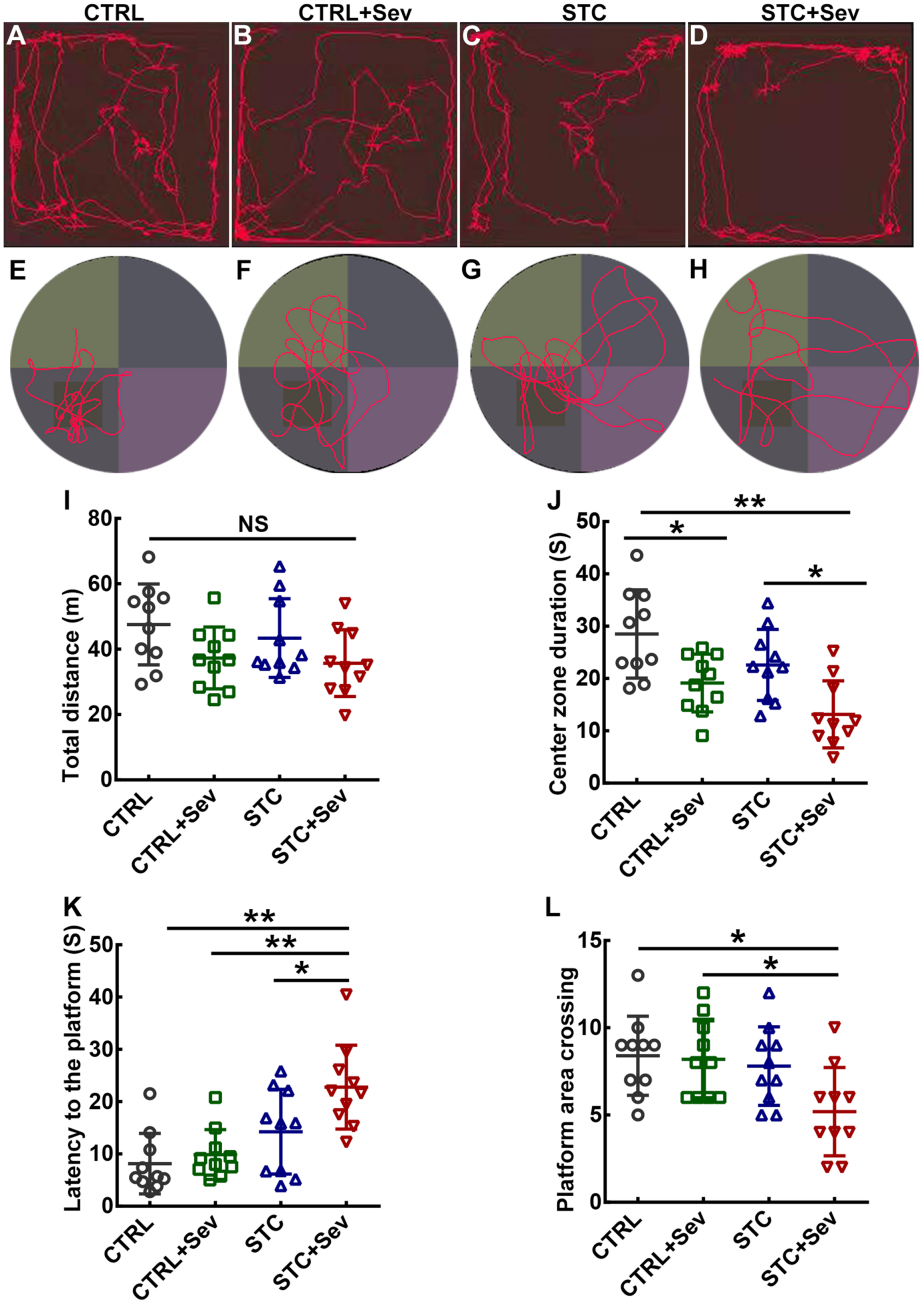
Sevoflurane exposure caused cognitive disorders and affective disorders in aged STC mice. **(A–D)** examples of recording trajectories in the open field test from the CTRL **(A)**, CTRL+Sev **(B)**, STC **(C)**, and STC + Sev **(D)** groups mice, respectively. **(E–H)** examples of recording trajectories in the Morris water maze test from the CTRL **(E)**, CTRL+Sev **(F)**, STC **(G)**, and STC + Sev **(H)** groups mice. **(I)** The distance traveled in the OFT (*n* = 10, F_3,16_ = 2.45, *p* = 0.08). **(J)** The time in the center zone in the OFT (*n* = 10, F_3,16_ = 8.72, *p* < 0.01). **(K)** The latency to the platform (*n* = 10, F_3,16_ = 9.18, *p* < 0.01). **(L)** Statistical analysis of the frequency of platform crossing in the probe trials (*n* = 10, F_3,16_ = 4.08, *p* < 0.01). One-way analysis of variance (ANOVA) with Tukey’s *post hoc* comparison. ^*^p < 0.05; ^**^p < 0.01. CTRL, control; Sev, sevoflurane exposure; STC, slow transit constipation.

Aberrantly activated microglia are the predominant source of inflammation and have been implicated in dysfunction damage and virtually all neurological diseases. Thus, to evaluate whether sevoflurane exposure caused neurobehavioral disorders in aged STC mice, we performed OFT and MWM. The trajectories of OFT and MWM in the four groups were depicted in Figures 5A–H, respectively. There were no significant differences in distance traveled between the four groups (Figure 5I, F_3,16_ = 2.45, *p* = 0.08). However, mice in the STC + Sev group spent significantly less time in the center zone compared with the STC and CTRL groups, and those in the CTRL+Sev group spent significantly less time in the center zone when compared with the CTRL group (Figure 5I, F_3,16_ = 8.72, *p* < 0.01). These data suggested that sevoflurane exposure caused anxiety-like disorders without spontaneous movement ability damage regardless of a state of constipation. Placed trials and probe trials of MWM were adopted to evaluate spatial learning and memory, respectively (25). In the placed trials of MWM, all mice could find the platform hidden under the water within 60 s and no significant differences in velocity were found between groups (data of velocity not shown). However, the mice in the STC + Sev group took, respectively, 2.8, 2.3, and 1.6 times as long as the CTRL, CTRL+Sev, and STC group mice to find the platform (Figure 5K, F_3,16_ = 9.18, *p* < 0.01). Consistently, the STC + Sev mice crossed the platform significantly less frequently compared to the CTRL and CTRL+Sev mice in the probe trials (Figure 5L, F_3,16_ = 4.08, *p* < 0.01).

## Discussion

The present study demonstrated that sevoflurane exposure caused increased gut permeabilities and intestinal injury in the aged mouse model of constipation. Gut-derived endotoxin leaking from the injured intestinal barrier resulted in systematic inflammatory responses and increased cytokines in circulation. Finally, activated microglia and the TLR4/NF-κB signaling pathway in the prefrontal cortex and hippocampus were associated with neurobehavioral changes.

STC is a common gastrointestinal disorder characterized by infrequent stools and/or difficult stool passage. The disorder impairs the quality of life in the elderly (26). As reported, μ-opioid receptor agonist loperamide caused spastic constipation by inhibition of intestinal peristalsis and resulting in prolonged retention of the intestinal contents in mice (27). In the present study, the STC model in aged mice was successfully established with loperamide intragastrically administrated for 7 days in aged mice as evidenced by defecation parameters of decreased fecal water content and prolonged time of first white pellet when compared to the control mice.

The intestinal barrier, the largest interface between the external environment and the internal milieu, includes surface mucus, epithelial layer, and immune defenses. However, apart from absorbing essential nutrients, the intestinal interface also confronts tremendous exterior antigens, microbiota, pathogens, and toxins (28). Thus, an intact intestinal barrier is crucial for maintaining mucosal homeostasis and protecting the organs against damage by microorganisms and toxins (29). Accumulating evidence demonstrated that intestinal permeability is tightly regulated by modulators including diet, gut microbiota modifications, mucus layer alterations, epithelial damage, hypoperfusion, toxins, and the enteric nervous system. In the present study, sevoflurane exposure caused significantly increased gut permeability 2 h after sevoflurane exposure and gradually decreased as evidenced by increased plasm concentration of 4-kDa FITC dextran in aged mice, which may be due to the rapid action and rate of elimination of sevoflurane. DAO is normally abundant in the mucosa or villi of intestinal mucosa but is rarely present in blood (30). After intestinal mucosa epithelial damage, the cytoplasm DAO can be released into the blood circulation. Thus, the increase of DAO in the serum reflects the damage to the mucosal integrity of the intestine, and its level in blood can be used to assess intestinal barrier function (30). Endotoxin, a lipopolysaccharide present in the cytoderm of Gram-negative bacteria, accumulates in the intestine lumen. Endotoxin can pass through the intestinal barrier and enter blood circulation after the intestinal barrier is injured, and its increase indicates bacteria and/or its endotoxin has translocated from the gut lumen to the circulatory system as a consequence of a dysfunctional intestinal barrier (31). Fatty acid binding proteins (FABPs) are small (14–15 kDa) cytosolic water-soluble proteins that are present in mature enterocytes of the intestine. FABPs transport fatty acids from the apical membrane to the endoplasmic reticulum of the enterocyte for biosynthesis of complex lipids. There are three types of FABPs in the gut, and intestinal FABP (I-FABP) is mainly presented in the jejunum, but less in the colon. I-FABP in the serum reflects the physiological turnover rate of enterocytes, whereas elevated levels indicate intestinal epithelial cell damage (29). Therefore, FITC-dextran, DAO, I-FABP, and LPS in the blood are ideal indicators reflecting the integrity and function of the intestinal barrier. We found that aged STC mice had significantly higher plasm levels of both DAO and LPS but not the I-FABP compared to the other three groups. These inconsistent changes might be due to their different peak time after injury induced by sevoflurane as we only tested one time point of 4 h after sevoflurane exposure and I-FABP may not have been detected at this time point. Nevertheless, our data revealed that the intestinal barrier and function are more susceptible to sevoflurane exposure.

In the present study, ten serum inflammatory cytokines were measured using a Bio-plex assay. Among these inflammatory cytokines, IL-2, IL-1, IL-5, IL-10, INF-γ, and TNF-α were significantly increased in the STC + Sev group compared to the CTRL, STC, and CTRL+Sev groups. Together with the data on gut permeability and intestinal barrier, we may conclude that aged mice exposed to sevoflurane had an increased gut permeability without detected systemic inflammatory response, whereas aged STC mice that experienced sevoflurane exposure showed increased gut permeability and higher levels of systemic inflammatory cytokines. Whether these changes were due to tight junction dysregulation, or temporary functional adjustment due to epithelial damage remains to be investigated. It has been reported that constipation is associated with microflora, increased intestinal permeability, and systemic immune response (17). Moreover, gut microbiota dysbiosis can affect gut permeability and is one of the pathogenesis of constipation (32, 33). Thus, gastrointestinal disorders such as chronic constipation might aggregate inflammatory response due to the “leaky gut” and microbiota dysbiosis, then consequently result in microbial translocation and systemic inflammatory response (28). Indeed, our and other previous studies reported that volatile anesthetics caused alterations in the composition of gut microbiota, which might be involved in the pathogenesis of postoperative cognitive dysfunction (34–36).

Preclinical studies also demonstrated that peripheral inflammation induced by anesthesia and surgery in combination can impair the structural and functional BBB integrity and increase inflammatory burden in the central nervous system (37, 38). Microglia, which constitute 5–10% of total brain cells, are resident cells of myeloid origin and serve as macrophages for innate immunity in the brain (39). The present study found that the number of activated microglia was significantly increased in the prefrontal cortex and hippocampus. Transmembrane proteins Toll-like receptors (TLRs) are a family of pattern recognition receptors (PRRs) of pathogen-associated molecular patterns (PAMPs). TLRs recognize diverse pathogen-associated molecules from bacteria, viruses, fungi, parasites, endotoxins, and interferon (IFN)-γ, contributing to the activation of the innate immune system. Among the TLRs, TLR4 is abundantly expressed in microglia (40). Up on the TLR4-dependent microglial activation and its downstream signaling cascades of latent transcription factors, particularly NF-κB, modulated microglia activation to produce and release pro-inflammatory mediators, including IL-1β, IL-6, TNFα, and reactive oxygen species (ROS), all of which then cause damage to the surrounding neuronal cells (41). Our data showed that TLR4 and NF-κB phosphorylation expressions were significantly upregulated and subsequently, in turn, caused the microglia activation.

The hippocampus and prefrontal cortex play a critical role in regulating multiple complex behaviors, including memory, cognition, attention, social interaction, and emotional regulation (14, 42). The dysfunction of these two brain regions is closely related to the clinical symptoms of POCD such as cognitive, attention, consciousness, and emotional changes (42–44). In the present study, we found that aged mice in the four groups studied displayed comparable levels of spontaneous locomotor activities in terms of distance traveled (Figure 5). In contrast, aged STC + Sev mice spent less time in the central area in the open field test. These findings suggest that aged STC + Sev mice exhibited different paradigms of emotional behavior in adapting to the environmental conditions compared to the other three groups. In addition, compared to the other three groups, aged STC mice spent more time locating the platform in the probe trials and registered a lower frequency of crossing the quadrant of the platform in the probe trials (Figure 5). These behavioral data suggested that aged STC mice displayed neurobehavioral deficits including impaired cognitive and abnormal emotional status, which might be due to the “leaky gut” and consequently promoted neuroinflammation in the brain.

There are some limitations in the present study. First, a surgical procedure such as orthopedic surgery was not introduced. Second, the BBB permeability and inflammatory cytokines were not assessed in the brain. Lastly, there are many elements involved in POCD but the behavioral tests designed in the present study are not enough to simulate POCD clinically. Hence, our study may be treated as a proof of concept and its translational value is subject to further study.

## Conclusion

In summary, the present study demonstrated that sevoflurane exposure caused increased gut permeability, intestinal injury, and higher levels of inflammatory cytokines, and consequently activated the TLR4/NF-κB signaling pathway and microglia in the key brain regions of aged STC mice. Furthermore, aged STC mice that were exposed to sevoflurane also had abnormal cognitive and affective behaviors. These results indicate that constipation may be a potential contributor to the development of POCD, and its prevention before and after surgery may be important for better recovery after surgery.

## Data availability statement

The original contributions presented in the study are included in the article/Supplementary material, further inquiries can be directed to the corresponding authors.

## Ethics statement

The animal study was approved by Animal Care and Use Committee of Jinan University, Guangzhou, China. The study was conducted in accordance with the local legislation and institutional requirements.

## Author contributions

XS and DM designed research. TZ, JL, JQ, YC, and WW performed experiments. ZS and PX analyzed data. TZ wrote the manuscript. DM critically revised the manuscript. All authors contributed to the article and approved the submitted version.

## Funding

This work was supported by grants from the National Natural Science Foundation of China (Grant no. 81870823, XS), Guangzhou Municipal Science and Technology Bureau (Grant no. 202201020597, WW), and Guangzhou Institute of Pediatrics/Guangzhou Women and Children’s Medical Center (Grant no. GCP-2018-001, XS; GCP-2019-002, TZ).

## Conflict of interest

The authors declare that the research was conducted in the absence of any commercial or financial relationships that could be construed as a potential conflict of interest.

## Publisher’s note

All claims expressed in this article are solely those of the authors and do not necessarily represent those of their affiliated organizations, or those of the publisher, the editors and the reviewers. Any product that may be evaluated in this article, or claim that may be made by its manufacturer, is not guaranteed or endorsed by the publisher.

## Figures and Tables

**Table 1:** 

BaSO4	Barium sulfate
BBB	blood–brain barrier
DAO	diamine oxidase
ELISA	Enzyme-Linked Immunosorbent Assay
ET	endotoxin
FABPs	Fatty acid binding proteins
Iba-1	ionized calcium-binding adaptor molecule 1
IFN	interferon
I-FABP	intestinal fatty acid-binding protein
LPS	lipopolysaccharide
mPFC	medial prefrontal cortex
MWM	Morris water maze
NF-κB	nuclear factor κB
OFT	Open field test
PAMPs	pathogen-associated molecular patterns
POCD	postoperative cognitive dysfunction
POD	postoperative delirium
PRRs	pattern recognition receptors
ROS	reactive oxygen species
Sev	sevoflurane
SD	standard deviation
STC	slow transit constipation
TLRs	Toll-like receptors
