# Supplementary figures and images for: Altered intestinal barrier contributes to cognitive impairment in old mice with constipation after sevoflurane anesthesia
Source: Front Nutr. 2023 Sep 12;10:1117028. doi: 10.3389/fnut.2023.1117028 (PMC10523324; doi:10.3389/fnut.2023.1117028)

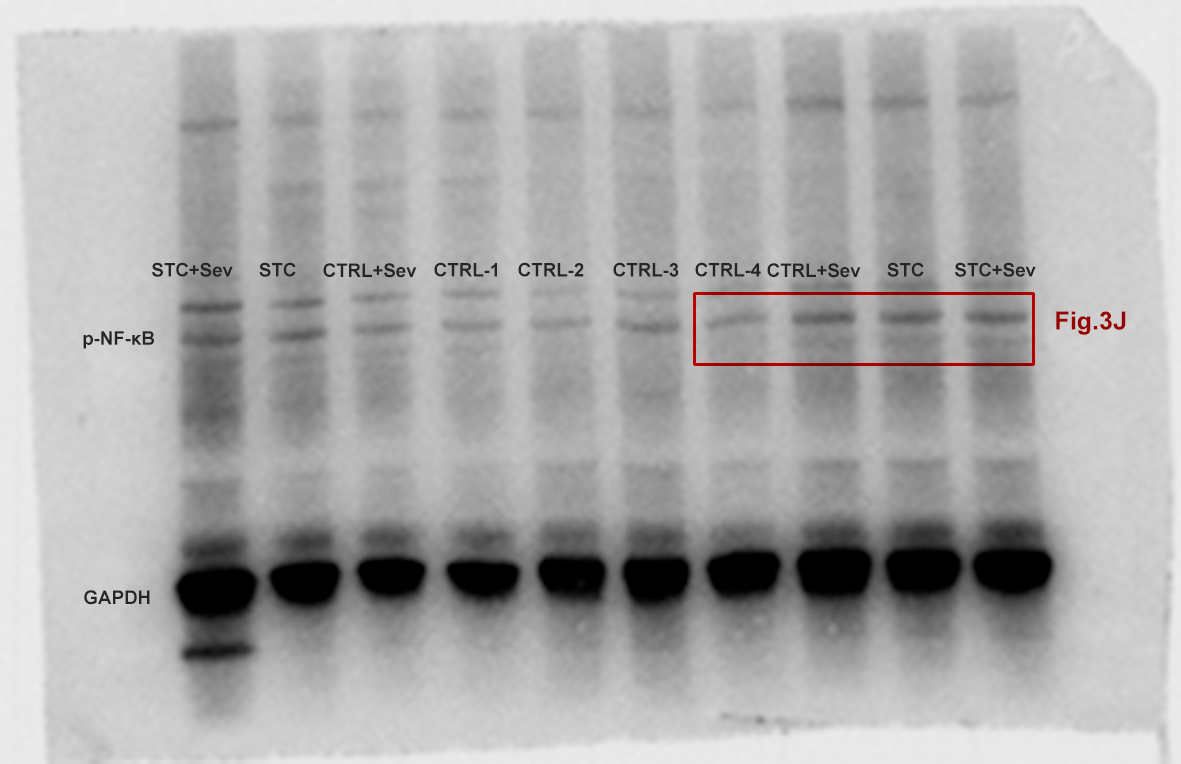

Supplement: Supplementary file 1 [file Image_1.TIF]

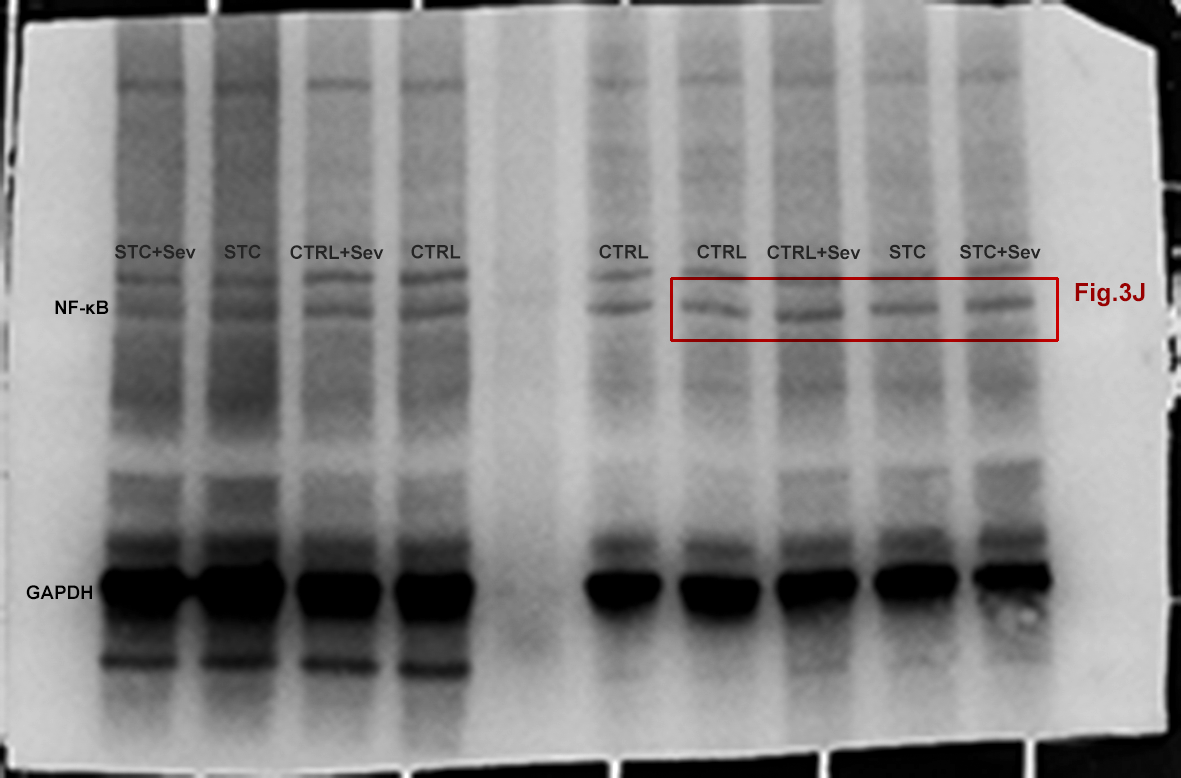

Supplement: Supplementary file 2 [file Image_2.TIF]
